# Structural Characterization and Anti-breast Cancer Activity *in vitro* of a Novel Polysaccharide From *Cymbopogon citratus*

**DOI:** 10.3389/fnut.2022.911838

**Published:** 2022-05-11

**Authors:** Yi Chen, Saifeng Qiao, Huiping Liu, Huizhen Xing, Pei Chen

**Affiliations:** State Key Laboratory of Food Nutrition and Safety, College of Food Science and Engineering, Tianjin University of Science and Technology, Tianjin, China

**Keywords:** *Cymbopogon citratus*, polysaccharide, structural characteristics, anti-breast cancer activity, Fas/FasL signaling pathway

## Abstract

*Cymbopogon citratus* is an important functional food, widely used for flavoring in Africa and South America. In this study, a novel high-molecular-weight polysaccharide (CCP) from *C. citratus* was extracted, and its structural characteristics and anti-breast cancer activity *in vitro* were investigated. CCP contained both α and β configurations and mainly composed of galactose (36.89%), arabinose (23.97%), glucose (18.35%) and rhamnose (9.36%) with an average molecular weight of 1.98 × 10^6^ Da. The main glycosyl residues of CCP detected by methylation analysis were 1,3,6-linked Gal*p*, 1,3-linked Glc*p*, 1,5-linked Ara*f* , T-Ara*f* , and T-Rha*p*. *In vitro* experiments suggested that CCP significantly inhibited the proliferation of MDA-MB-231 cells, decreased the expressions of cyclin D1 and CDK4 and stocked cells at G_0_/G_1_ phase. Meanwhile, the typical morphological features of apoptotic cells were also observed. Combining with the consequences of Annexin V-FITC/PI staining, Hoechst 33258 staining and western blot analysis, CCP induced apoptosis of MDA-MB-231 cells by triggering the Fas/FasL-mediated death receptor pathway. Overall, these results provide a theoretical basis for the application of *C. citratus* polysaccharide as a potential anti-breast cancer agent in functional food and medicine.

## Introduction

Malignant tumors are considered to be a key threat to human health worldwide due to their high recurrence rate and high mortality (1). Among cancer subtypes, breast cancer (BC), which accounts for almost 30% of all female cancers worldwide, is the second most prevalent cancer and the fifth leading cause of death from cancer in women (2–4). Nowadays, chemotherapy is regarded as the most commonly used in the clinical treatment of BC, improving the survival rate of patients (5). However, the clinical use of chemotherapy agents such as anthracyclines is frequently associated with severe adverse reactions, like acute and chronic dose-dependent cardiotoxicity (6, 7). Accumulating evidence indicates that natural products extracted from plants possess strong antitumor activity and had no significant adverse effects compared with synthetic compounds (8, 9). Hence, it is necessary to seek natural products with high efficacy and low toxicity for BC treatment.

Polysaccharides are widely existing in animals, plants and microorganisms, and have various physiological activities such as antioxidant, antiviral, hypoglycemic, anti-tumor and immunomodulatory (10–12). *Cymbopogon citratus* (lemongrass), belonging to the genus *Cymbopogon*, is widely cultivated in tropical countries, especially in Southeast Asia (13). *Cymbopogon citratus* has been used in the food, perfume, soap, cosmetics, pharmaceutical and pesticide industries (14). For many years, the biological properties of *C. citratus* have been reported, including but not limited to anti-inflammatory, antibacterial, antiprotozoal, antitussive, antioxidant, anticancer, cardioprotective and antirheumatic activities (15–17). Studies have shown that *C. citratus* contains polyphenols, terpenes, alcohols, ketones, citral and polysaccharides (18, 19). Recently, the research of *C. citratus* polysaccharide has attracted the attention of scholars. It has been researched that polysaccharide is one of the main active ingredients of *C. citratus* and possesses a variety of bioactivities, such as preventing neurodegenerative diseases, immuno-stimulating activity, anti-oxidant and anti-tumor activity (20–22).

*Cymbopogon citratus* extract has been shown to have anti-breast cancer effect (23, 24). However, there are few reports on the efficacy of *C. citratus* polysaccharide in breast cancer cell lines, and the mechanism of its anti-breast cancer cells has not been reported. Therefore, a novel water-soluble polysaccharide (CCP) from *C. citratus* was prepared and the mechanism of its action on MDA-MB-231 cells was studied. This study highlights the potential of CCP in functional foods for breast cancer therapy, and provide new ideas for the development and utilization of *C. citratus* in the food and pharmaceutical industry.

## Materials and Methods

### Materials and Reagents

The leaves of *C. citratus* was purchased from Chinese herbal medicine market in Anguo (Anguo, China). Human breast cancer MDA-MB-231 cells were obtained from Shanghai Cell Bank of Chinese Academy of Sciences (Shanghai, China). The 0.25% trypsin and fetal bovine serum (FBS) were bought from GIBCO (Carlsbad, USA). The 3-(4,5-Dimethyl-2-thiazolyl)-2,5-diphenyl-2-H-tetrazolium bromide (MTT), Hoechst 33258, Propidium Iodide (PI), Annexin V-FITC/PI apoptosis detection kit and Reactive Oxygen Species assay kit were purchased from Solarbio Science & Technology Co. Ltd (Beijing, China). Bradford protein assay (BCA) kit and enhanced chemiluminescence (ECL) detection kit were provided by Beyotime Biotechnology (Shanghai, China). All antibodies were obtained from Wuhan Sanying Biotechnology (Wuhan, China). All of other chemicals and reagents were analytical grade.

### Isolation and Purification of the *Cymbopogon citratus* Polysaccharide

The dried *C. citratus* leaves were pulverized to obtain the powder. And the powder was extracted with ultrapure water (solid-liquid ratio of 1:20, w/v) at 80°C for three times (each time for 2 h). The aqueous extracts were concentrated with a rotary evaporator to 1/3 of the original volume, and precipitated *via* adding absolute ethanol (final concentration 60%) at 4°C for 24 h. The precipitate was collected and dissolved in water, then added with Sevag solution to remove the protein. Subsequently, the polysaccharide solution was dialyzed to remove small molecular substances, and lyophilized to obtain the crude polysaccharide. Finally, we further purified the crude polysaccharide through Sephadex G-200 column, and then reserved after freeze-drying, named as CCP.

### HPGPC Assays

The molecular weight of CCP was calculated according to the standards T-series dextrans (T-10, T-40, T-70, T-500 and T-2000), which using a high-performance gel permeation chromatograph (HPGPC; Agilent-1200, USA) equipped with a Tsk-gel G4000PWxl column (7.8 × 300 mm, column temperature 30°C) and refractive index detector (RID, detecting temperature 35°C; Schambeck SFD GmbH, Bad Honnef, German). Twenty microliter CCP solution (1 mg/ml) was injected by using ultrapure water as the mobile phase at flow rate of 0.6 ml/min.

### UV–Visible Spectra and FT-IR Spectrum Assays

Using distilled water as a reference, 1 mg/ml CCP solution was scanned at the range of 200–400 nm by UV-vis spectrophotometer (spectrum-2102UV, USA). The FT-IR spectrum was scanned by KBr tablet method using Fourier-transform infrared spectrophotometer (Bruker VECTOR-22, Germany) in the range from 4,000 to 400 cm^−1^.

### Monosaccharide Composition and NMR Analysis

Ion chromatography (IC; Dionex ICS2500, USA) was used to investigate the monosaccharide composition of CCP. CCP was degraded for 3 h at 110°C after dissolved by using trifluoroacetic acid (TFA). After hydrolysis, the excess TFA was removed by the addition of methanol and blow-dried three times with nitrogen (N_2_). Then we dissolved the hydrolysis with ultrapure water completely and diluted to 200 ppm. Meanwhile, L-fucose (Fuc), L-rhamnose (Rha), L-arabinose (Ara), D-galactose (Gal), D-glucose (Glc), D-xylose (Xyl), D-mannose (Man), D-ribose (Rib), D-glucuronic acid (GlcA) and D-galacturonic acid (GalA) were prepared as references. The CCP was dissolved in D_2_O, and detected with the Bruker Advance DPX-500 spectrometer to obtain the ^1^H NMR and ^13^C NMR spectra.

### Methylation Analysis

CCP was methylated according to the reported method with some modifications (25, 26). Ten milligram of dry CCP powder was weighed and dissolved in dimethyl sulfoxide (DMSO). After adding NaOH, the reaction was carried out under ultrasonic and dark conditions. Then the solution was mixed with iodomethane and kept at the same condition. After the reaction, water and dichloromethane were added, and the dichloromethane phase was collected and dried. The methylated products were analyzed by FT-IR. The methylation sample was hydrolyzed with trifluoroacetic acid (TFA) at 121°C, and subsequently was dried. The ammonium hydroxide and NaBD_4_ were added and allowed to react at room temperature. The reaction was stopped by adding the acetic acid and drying them under nitrogen flow. The reaction product was then gently washed with methanol and subsequently acetylated using acetic anhydride at 100°C. After adding water and dichloromethane, the water phase was discarded and the dichloromethane phase was collected and analyzed using GC-MS (7890A-5977B, Agilent Technologies Inc., CA, USA). The split ratio was 10:1 and Helium was used as carrier gas. The temperature programed was set at 140°C for 2 min initially with gradually increasing at 3°C/min to 230°C, finally kept at 230°C for 3 min.

### Cell Culture

DMEM with 10% FBS was used to culture MDA-MB-231 cells. These cells were kept in an incubator (Thermo, USA) with 95% humidity and 5% CO_2_ at 37°C.

### MTT Assay

The viability of MDA-MB-231 cells was determined by MTT assay. 5 × 10^4^ cells per well were seeded in 96 well plates. After 48 h of treatment with CCP, MTT (5 mg/ml, 20 μl) was added to each well incubated for 4 h. Then, 150 μl of DMSO was added to each well with vibration until the insoluble purple crystal product was dissolved. The absorbance values of each well were recorded at 570 nm by Microplate Reader (Bio-Rad, USA). The growth inhibitory rate formula is as follows:


$$\begin{array}{l} \text{Inhibitory rate }\left(\%\right)=\left(1-\frac{\text{B}}{\text{A}}\right)\times 100 \end{array}$$

where A is the absorbance value of control group, B is absorbance value of CCP treatment group.

### Cell Morphological Observation

The MDA-MB-231 cells, with a density of 2 × 10^5^ cells/ml, were incubated in six well plates. The morphological changes were observed under inverted light microscope (Nikon, Japan) after the cells were treated with distinct levels of CCP for 48 h. The nuclear morphological changes were evaluated by Hoechst 33258 staining. Cells cultured with CCP were washed three times with phosphate buffered saline (PBS). Then 1 ml of Hoechst 33258 was added to cells followed by washing with PBS. The changes of nuclear morphology in untreated and treated groups were observed by fluorescence microscope (Nikon, Japan).

### Cell Apoptosis Assay

The apoptosis rate of MDA-MB-231 cells was detect using Annexin V-FITC/PI staining. The treated cells were digested with trypsin and collected in centrifuge tube. Then, the precipitate was washed three times with PBS. According to the instructions of the apoptosis detection kit, the Annexin V-FITC and PI were added to suspension cells and incubated for 10 min at the room temperature without light. After that, the flow cytometry (Becton Dicknson, USA) was used to measure the stained cells.

### Measurement of Intracellular ROS

The 2,7-Dichlorodihydrofluorescein diacetate (DCFH-DA) staining was applied to measure the production of intracellular ROS. Cells treated with CCP (0, 400, 600 and 800 μg/ml) for 48 h were collected, and centrifuged to remove the supernatant. Then the precipitate was incubated with 20 μl DCFH-DA at 37°C for 20 min. After incubation, ROS was immediately analyzed with a flow cytometry (Becton Dicknson, USA).

### Cell Cycle Assay

Propidium Iodide staining was performed to measure dose-response effects of CCP on cell cycle progression. MDA-MB-231 cells treated with distinct levels of CCP were harvested and fixed with precooled 70% ethanol solution at 4°C for 18 h, respectively. After that, the fixed cells were incubated with 0.1 mg/ml RNase A and stained with 50 μg/ml PI for 10 min at 37°C. Finally, the stained cells were analyzed by a flow cytometry (Becton Dicknson, USA).

### Western Blot Assay

The apoptosis-related proteins expression was detected by Western blot assay. Cell lysates were prepared at 4°C by adding phenylmethylsulfonyl fluoride (PMSF) inhibitor into RIPA lysis buffer. 12% sodium salt (SDS)-Polyacrylamide gel electrophoresis (SDS-PAGE) was used to separate the protein samples, and then it was imprinted on PVDF membranes. TBST buffer containing 5% bovine serum albumin was used to block the membrane for 2 h. The membrane was incubated with primary antibody overnight at 4°C, which contained β-actin, cyclin D1, CDK4, FasL, Fas, FADD, caspase-3, caspase-8. Subsequently, the horseradish peroxidase conjugated antibodies were incubated with the washed membranes for 2 h at room temperature. Ultimately, the protein bands were detected using ECL reagent in a dark room. Then, the densitometric quantification was carried out using ImageJ Software.

### Statistical Analysis

All data were expressed as mean ± standard deviation (SD) and the analysis was evaluated using GraphPad Prism version 6. Student's *t*-test was used to evaluate the significant differences between two groups. ^*^P < 0.05 compared to control group was considered as significant. ^**^P < 0.01 compared to control group was considered as very significant. ^***^P < 0.001 compared to control group was considered as extremely significant.

## Results

### Molecular Weight and UV Analysis of CCP

**Figure 1:**
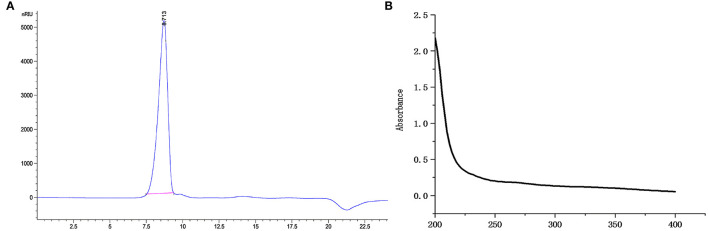
The HPGPC chromatograms **(A)** and UV-vis spectra **(B)** of CCP.

CCP was isolated from *C. citratus* through hot-water extraction, ethanol precipitation, deproteinization by Sevage, dialysis against water and Sephadex G-200 gel column purification. The yield of CCP was 3.42 ± 0.31%. Determination of the molecular weight of CCP using HPGPC. A single narrow symmetrical peak with a retention time of 8.713 min was observed (Figure 1A), indicating that CCP was a homogeneous high-molecular-weight polysaccharide. According to the regression equation (*y* = −0.3967*x* + 9.7523, *R*^2^ = 0.9985), the molecular weight of CCP is 1.98 × 10^6^ Da. CCP had no obvious absorption peak at 260~280 nm (Figure 1B), which indicated that it contains almost no protein or nucleic acid (27).

### FT-IR Analysis and Monosaccharide Composition of CCP

**Figure 2:**
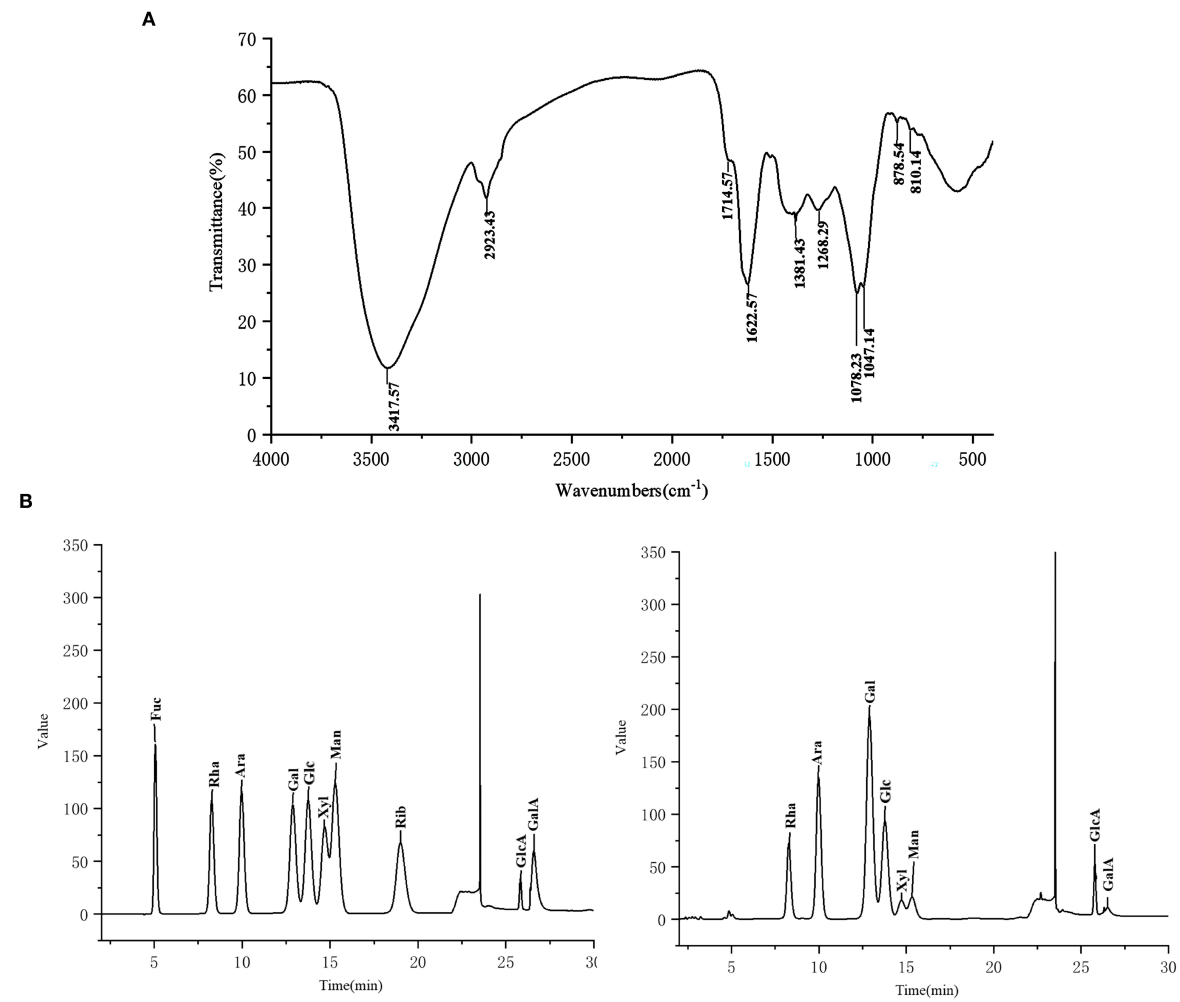
The FT-IR spectra **(A)** and monosaccharide composition **(B)** of CCP.

FT-IR spectra of CCP was displayed in Figure 2A. The characteristic absorption peaks of polysaccharide appeared at 3,417.57, 2,923.43, 1,381.43 and 1,268.29 cm^−1^, respectively, due to the stretching vibration of –OH, the stretching, the variable angle and the bending vibrations of C–H (28). The absorption peak at 1,622.57 cm^−1^ was obtained, which derived from C=O stretching vibration (29). There was a weak absorption peak at 1,714.57 cm^−1^ showed the presence of lesser amounts of uronic acid in CCP. And the bands ranging from 1,000 to 1,200 cm^−1^ were attributed to the vibration of C–O–C glycoside ring, which suggested the presence of pyranose (30). Additionally, the peaks at 810.14 and 878.54 cm^−1^ confirmed that CCP contained α- and β- glycosidic bonds (31). The IC results (Figure 2B) showed that CCP was mainly composed of Rha, Ara, Gal and Glc, but also contained a small amount of Xyl, Man, Glca and GalA. Moreover, the molar ratio of Rha, Ara, Gal, Glc, Xyl, Man, Glca and GalA was 1:2.56:3.94:1.96:0.41:0.42:0.29:0.10.

### NMR Analysis of CCP

**Figure 3:**
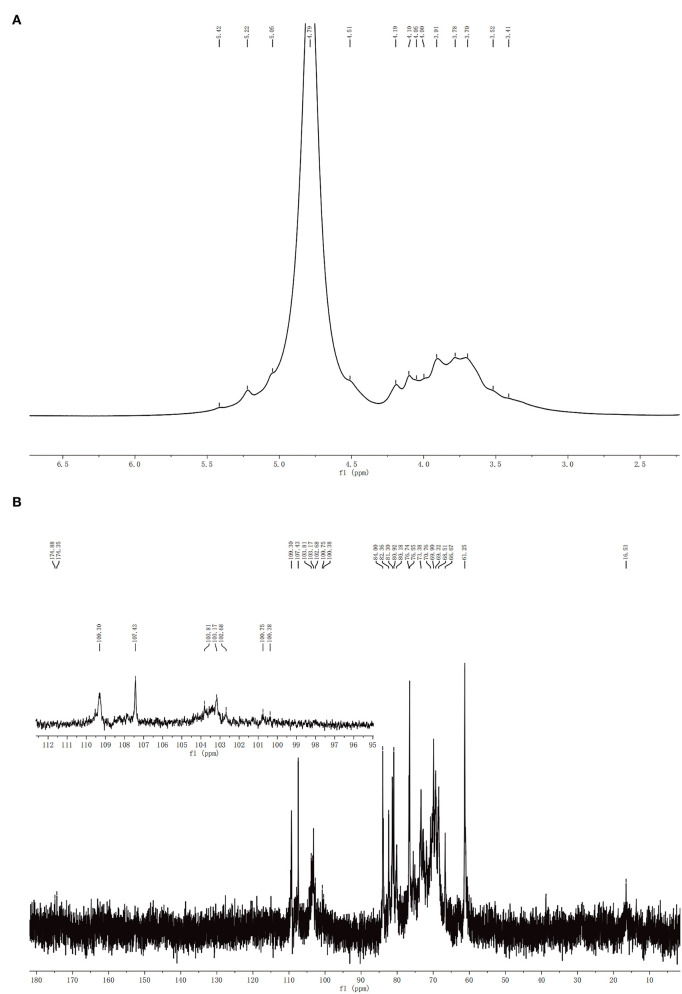
^1^H **(A)** and ^13^C **(B)** NMR spectra of CCP.

^1^H NMR and^13^C NMR spectra of CCP were showed in Figure 3. Usually, the chemical shift of the α-form was observed at δ5.0–6.0 ppm, whereas the chemical shift of the β-form was δ4.5–5.0 ppm (32). It can be seen that CCP had the chemical shifts in both segments, which determined that α- and β-configurations were present. In ^1^H NMR spectrum, the peak overlap of spectrum signal was serious and it was not suitable for attribution. There were multiple signal peaks in the 13C NMR spectrum from 90 to 110 ppm, indicating that CCP contained both α-glycosidic and β-glycosidic bonds, which was consistent with the ^1^H NMR conclusion. The signal at 16.53 ppm was assigned to α-L-Rha. The peaks in the region of δ110–100.38 ppm indicated the presence of α-L-Ara, β-D-Gal, β-D-Glc, α-D-Man, α-D-Gal, and β-D-Xyl (33, 34). The ^13^C chemical shifts of CCP between 170 and 180 ppm were attributed to the presence of a small amount of uronic acid. The results of NMR analysis were consistent with those of monosaccharide composition analysis and FT-IR analysis.

### Methylation Analysis of CCP

The glycosidic linkages of the CCP were determined by methylation analysis. On the basis of the monosaccharide composition of CCP, the glycosyl linkages of the monosaccharides are presented and summarized in Table 1 according to the retention times and mass fragments in GC-MS. As summarized in Table 1, CCP was composed of nine glycosidic residues assigned to be 1,3,6-linked Gal*p* (24.36%), 1,3-linked Glc*p* (18.33%), 1,5-linked Ara*f* (15.26%), T-Rha*p* (10.67%), T-Ara*f* (9.76%), 1,6-linked Gal*p* (8.03%), 1,6-linked Man*p* (4.62%), 1,3-linked Gal*p* (4.51%), and 1,4-linked Xyl*p* (4.49%). The proportions of Gal, Ara, Glc, Rha, Man and Xyl (36.90:25.02:18.33:10.67:4.62:4.49) was basically close to the result of monosaccharide composition, which indicate the reliability of methylation analysis.

**Table 1:** Methylation analysis of CCP.

**Retention time**	**Linkage type**	**Methylated sugars**	**Molar ratio (%)**	**Mass fragments (*m*/*z*)**
5.976	T-Rha*p*	1,5-di-O-acetyl-6-deoxy-2,3,4-tri-O-methyl rhamnitol	10.67	59, 72, 89, 102, 118, 131, 145, 162, 175, 211
6.243	T-Ara*f*	1,4-di-O-acetyl-2,3,5-tri-O-methyl arabinitol	15.26	71, 87, 102, 118, 129, 145, 161
10.965	5-Ara*f*	1,4,5-tri-O-acetyl-2,3-di-O-methyl arabinitol	9.76	59, 87, 102, 118, 129, 162, 189, 232
11.806	4-Xyl*p*	1,4,5-tri-O-acetyl-2,3-di-O-methyl xylitol	4.49	71, 87, 103, 118, 129, 149, 162, 189, 205
12.573	3-Glc*p*	1,3,5-tri-O-acetyl-2,4,6-tri-O-methyl glucitol	18.33	59, 87, 101, 118, 129, 161, 202, 234
13.264	3-Gal*p*	1,3,5-tri-O-acetyl-2,4,6-tri-O-methyl galactitol	4.51	59, 87, 101, 118, 129, 161,174, 243
14.154	6-Man*p*	1,5,6-tri-O-acetyl-2,3,4-tri-O-methyl mannitol	4.62	71, 87, 99, 102, 118, 129, 162, 189, 233
15.906	6-Gal*p*	1,5,6-tri-O-acetyl-2,3,4-tri-O-methyl galactitol	8.03	71, 87, 99, 102, 118, 129, 162, 189, 233
19.389	3, 6-Gal*p*	1,3,5,6-tetra-O-acetyl-2,4-di-O-methyl galactitol	24.36	59, 87, 101, 118, 129, 160, 189, 234

### CCP Inhibited the Cells Proliferation

**Figure 4:**
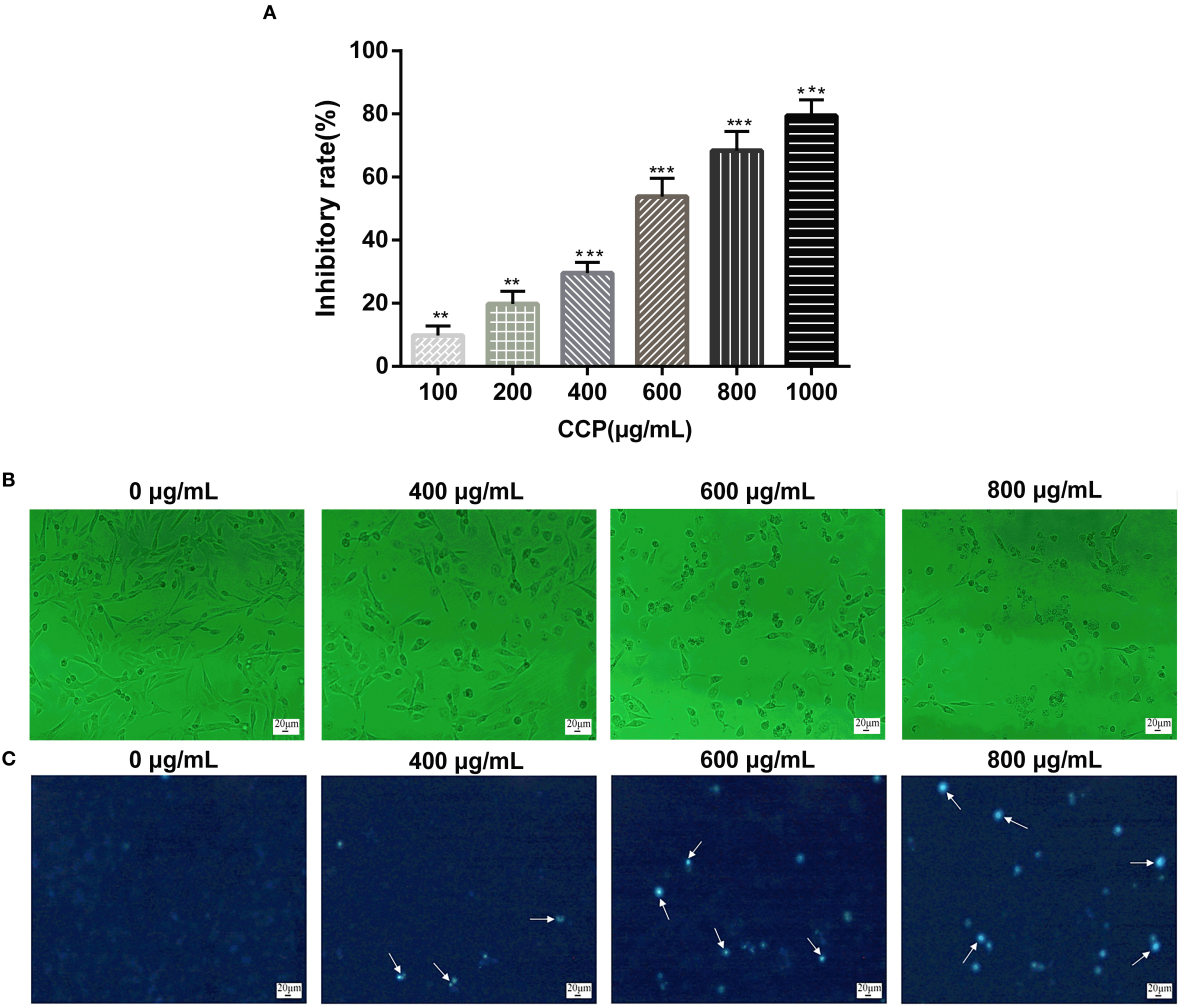
**(A)** Effect of CCP on MDA-MB-231 cells proliferation. The morphological characteristics **(B)** and nuclear morphological changes **(C)** of cells treated with CCP. The white arrows represent apoptotic nuclear fragments. **P < 0.01 and ***P < 0.001 vs. control group.

The cell growth inhibitory rate was assessed by MTT assay to determine the cancer suppressive potential of CCP *in vitro*. The experimental data (Figure 4A) suggested that CCP remarkably suppressed MDA-MB-231 cell proliferation in the dose-dependent manner. The inhibition rate of MDA-MB-231 cells treated with CCP (600 μg/ml) for 48 h was approximately 50%. Therefore, MDA-MB-231 cells were treated with 400, 600, and 800 μg/ml CCP for 48 h in subsequent experiments, respectively. The results demonstrated that CCP could act as a potential anticancer drug.

### CCP Induced Cells Morphological Changes

The morphological changes in MDA-MB-231 cells on treatment with CCP were presented in Figure 4B. The normal MDA-MB-231 cells displayed well growth, typical spindle shapes and clear edge. However, after treated with CCP in different concentrations for 48 h, the cells exhibited apparent morphological alterations including cells volume reduction, cells surface curl and cytoplasmic vacuoles appearance. Especially, most of the cell membranes seriously contracted, the number of exfoliated cells gradually increased, apoptotic bodies gradually appeared and some cells were broken into particles of different sizes.

The morphological characteristics changes of nucleus of apoptotic cells were further investigated by Hoechst 33258 staining (Figure 4C). In the untreated group, the nucleus was light blue, indicating a stable distribution of chromatin in the nucleolus. As chromatin aggregates and nucleoli contract, the treated cell glows bright blue fluorescence and the intensity of bright blue fluorescence also increased with the increase of CCP concentration. The staining results were consistent with the results of morphological observation. These phenomena indicated that CCP induces apoptosis in MDA-MB-231 cells.

### CCP Induced Cells Apoptosis

**Figure 5:**
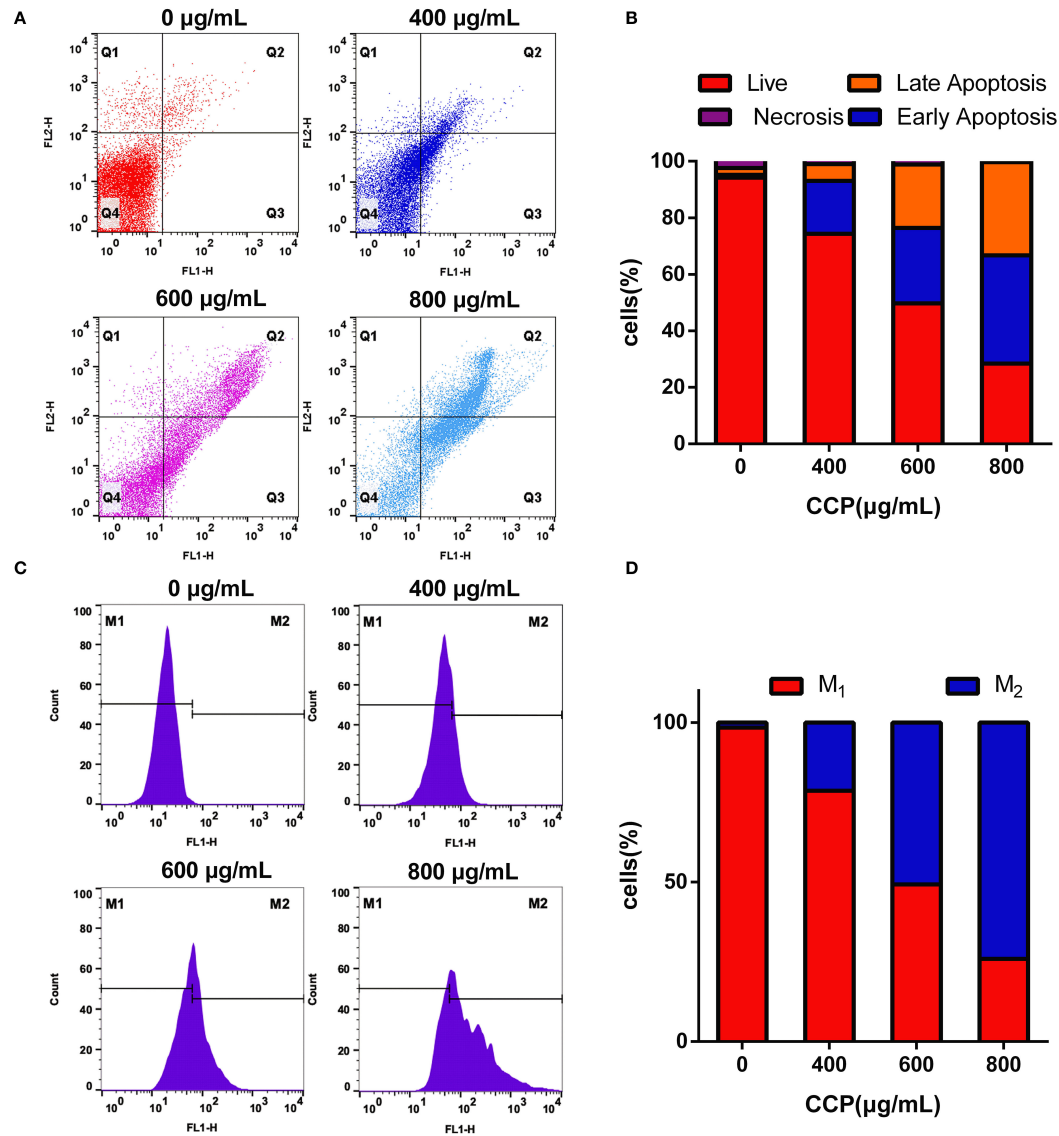
The scatter **(A)** and column **(B)** showed the Annexin V-FITC/PI staining results. The DCFH-DA staining results **(C)** and quantitative analysis **(D)** of the intracellular ROS.

Apoptosis cells induced by different concentrations of CCP were further confirmed by Annexin V-FITC/PI double staining (Figure 5A). The viable cells decreased gradually and apoptotic cells increased significantly in the experimental group in a dose-dependent manner. With the increase of CCP concentration, the early apoptotic cells up-regulated from 1.02 to 38.31% and the late apoptotic cells elevated from 2.42 to 33.07%, while the percentage of alive cells was reduced from 94.20 to 28.46% (Figutre 5B). These results con-firmed that CCP had the significant effect on regulating MDA-MB-231 cells apoptosis.

Apoptosis has been increasingly proved to be closely associated to oxidative stress (35). The effect of CCP on production of intracellular ROS was analyzed by flow cytometry. Compared with the untreated group (Figure 5C), the fluorescence intensity of CCP-treated cells was significantly enhanced in a dose-dependent manner. The level of ROS increased from 1.18 to 25.10%, 51.34 and 74.47%, respectively. It is verified that CCP may induce MDA-MB-231 cells apoptosis *via* triggering ROS generation.

### CCP Induced Cells Cycle Arrest

**Figure 6:**
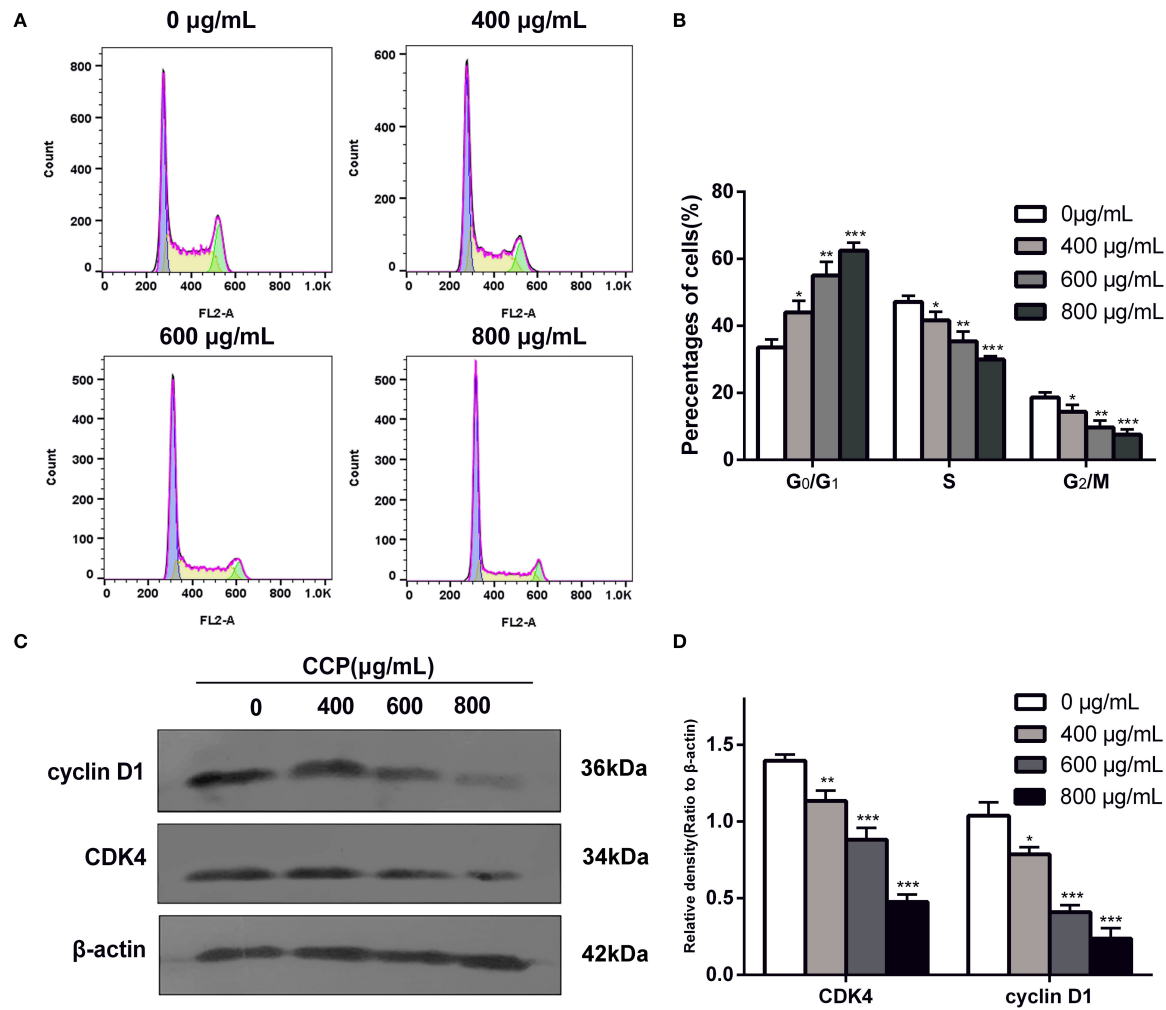
Distribution **(A)** and proportions **(B)** of treated cell in various phases of the cell cycle. The expressions levels **(C)** and quantitative analysis **(D)** of cyclin D1 and CDK4. *P < 0.05, **P < 0.01 and ***P < 0.001 vs. control group.

Changes in DNA content were detected by flow cytometry to assess the proportional distribution of cells throughout the cell cycle. Results (Figures 6A,B) showed that after treatment with different CCP concentrations (0, 400, 600, 800 μg/ml), the G_0_/G_1_ phase of MDA-MB-231 cells increased from 35.73 to 44.10%, 55.21 and 60.43%, respectively. Meanwhile, the significant reduction in the percentage of cells in the G_2_/M phase and S phase were found. As the proteins associated with the G_0_/G_1_ phase, cyclin D1 and CDK4 were detected using western blot analysis to further investigate the G_0_/G_1_ phase arrest. The experimental data suggested that cyclin D1 and CDK4 levels were significantly decreased as the concentration of the CCP increased (Figures 6C,D). These data suggested that CCP induced G_0_/G_1_ phase cycle arrest in MDA-MB-231 cells.

### CCP Regulated the Expression of Apoptosis-Associated Proteins

**Figure 7:**
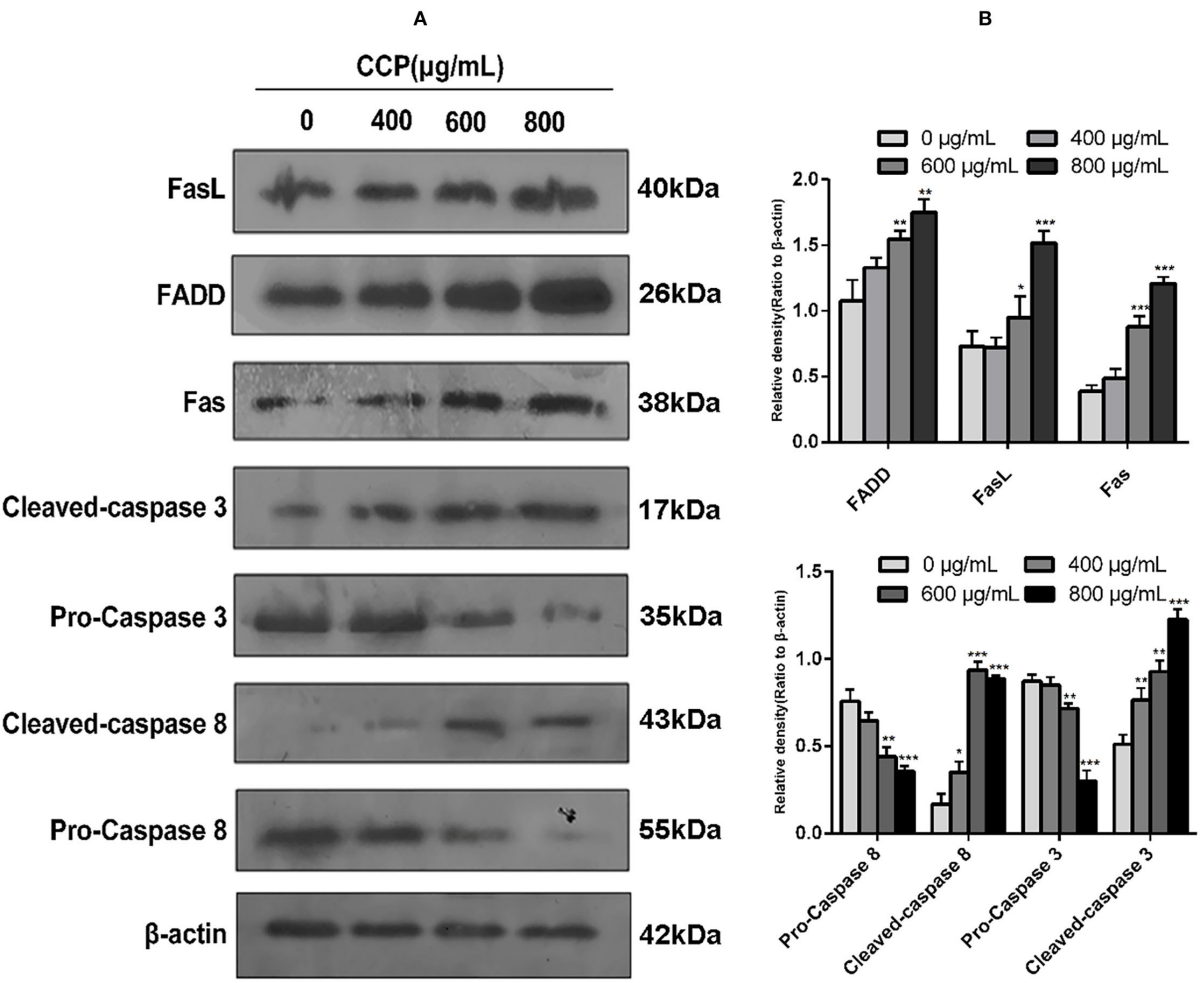
**(A)** Western blot assay for FasL, FADD, Fas, caspase-3, cleaved-caspase-3, caspase-8 and cleaved-caspase-8 protein expression levels. **(B)** Columns represent the expression of the FasL, FADD, Fas, caspase-3, cleaved-caspase-3, caspase-8 and cleaved-caspase-8 relative to β-actin. *P < 0.05, **P < 0.01 and ***P < 0.001 vs. control group.

To confirm the role of the Fas/FasL death pathway in CCP-induced apoptosis of MDA-MB-231 cells, we determined the protein expressions of Fas, FasL, FADD, Caspase-3 and Caspase-8. The results (Figure 7) showed that CCP treatment resulted in a dose-dependent increased in the level of FADD, Fas and FasL. And the expression of pro-caspase-8 was decreased in CCP-treated MDA-MB-231 cells, while the expression of cleaved-caspase-8 was increased. These indicated that caspase-8 was activated. Subsequently, the cleaved-caspase-3 level in treated cells significantly increased, which confirmed that caspase-3 was activated downstream of caspase-8. These results suggest that CCP played its pro-apoptotic role in MDA-MB-231cells by regulating the Fas/FasL pathway.

## Discussion

In this study, a novel water-soluble polysaccharide (CCP) was extracted from *C. citratus*. The molecular weight of CCP (1.98 × 10^6^ Da) was much larger than those reported in previous studies, which are hinge on extraction and purification conditions (36). It has been suggested that the high-molecular-weight polysaccharides shown superior antitumor activity (37). Although we suspect that the antitumor activity of CCP may be related to the ability of high-molecular-weight polysaccharides to form higher-order structures, this will need further research. It is worth noting that the monosaccharide composition of *C. citratus* polysaccharides shows a diversity distribution, some are mainly composed of Glc, and some consists mainly of Xyl, which is different from the monosaccharide composition of CCP mentioned in this experiment (36, 38). The content of galactose in CCP was higher than the other monosaccharides such as Glc, Xyl, Ara, Rha and Man. Most of the present studies on the antitumor efficiency of *C. citratus* polysaccharide are limited to crude polysaccharides and low-molecular-weight polysaccharides, and little information about the anti-breast cancer activity of polysaccharides from this plant is available. Therefore, this study examined structural characteristics and the anticancer effect of extracted polysaccharide with high molecule weight on MDA-MB-231 cells and further investigated the action mechanism. To the best of our knowledge, this is first time that high-molecular-weight polysaccharide from *C. citratus* induced G_0_/G_1_ arrest and apoptosis of MDA-MB-231 cells *via* Fas/FasL-mediated death receptor pathway is reported.

Inhibition of cancer cell proliferation is an effective way to promote cancer cell death. In this experiment, MTT assay revealed that CCP had a dose-dependent inhibitory effect on the proliferation of MDA-MB-231 cells. Apoptosis is considered to be the programmed physiological death of cells, which is related to homeostasis, physiology and pathology (39). Cell death caused by apoptosis involved multiple particular morphological changes, such as cell volume reduction, chromatin condensation, cytoplasmic vacuolation, nucleolus fragmentation of the nuclear membrane, formation of apoptotic bodies, and extravagation of phosphatidylserine (PS) inside the membrane to the membrane surface (40). Annexin-V, a phospholipid binding protein, has higher affinity for PS and can combine with the nucleic acid dye PI to distinguish viable cells from apoptotic and necrotic cells (40, 41). The characteristic morphological changes of the MDA-MB-231 cells were obviously observed in this study when they were treated with CCP for 48 h. In addition, CCP had been further demonstrated to induce apoptosis of MDA-MB-231 cells according to the Annexin V-FITC/PI double staining results. Reactive oxygen species (ROS), containing hydroxyl radicals and singlet oxygen, are closely as-sociated with oxidative stress. According to Parvaiz, ROS was involved in the process of protein fraction from *Withania somnifera* induced apoptosis of MDA-MB-231 cells (42). Our findings indicated that different concentrations of CCP significantly induced ROS production in MDA-MB-231 cells, which may induce cell apoptosis.

Since cell cycle can regulate cell proliferation, differentiation and apoptosis, interfering with cell cycle is recognized as a potent approach for cancer treatment (43). The realization of cell cycle progression depends on the precise and rigorous regulation of cell cycle by various levels of regulatory factors, such as cyclin-dependent kinases (CDK) and cyclins (44). It is known that many substances could modulate regulators at specific cell cycle checkpoints to provoke cell cycle arrest (45–47). Cyclin D1/D3 and Cyclin E were closely related to CDK4/6 and CDK2, which played a pivotal role in controlling the progression of G_1_ cell cycle (48). Li et al. (49) found that a heptamethine cyanine dye (IR-783) decreased the levels of cyclin D1, cyclin E, CDK2 which led to cycle G_0_/G_1_ arrest in breast cancer cells. Likewise, Gao et al. (50) pointed out that β-Cryptoxanthin mediated the downregulation of cyclin E, cyclin D1, and CDK4, CDK6 to induce G_0_/G_1_ arrest in SGC-7901 cells and AGS cells. In this study, flow cytometry detected that cell cycle was strongly arrested at G_0_/G_1_ phase. Consistent with this, we observed significant de-creased levels of cyclin D1 and CDK4 in MDA-MB-231 cells treated with CCP. These results indicated that cell cycle of MDA-MB-231 cells was arrested at G_0_/G_1_ phase in the case of CCP.

**Figure 8:**
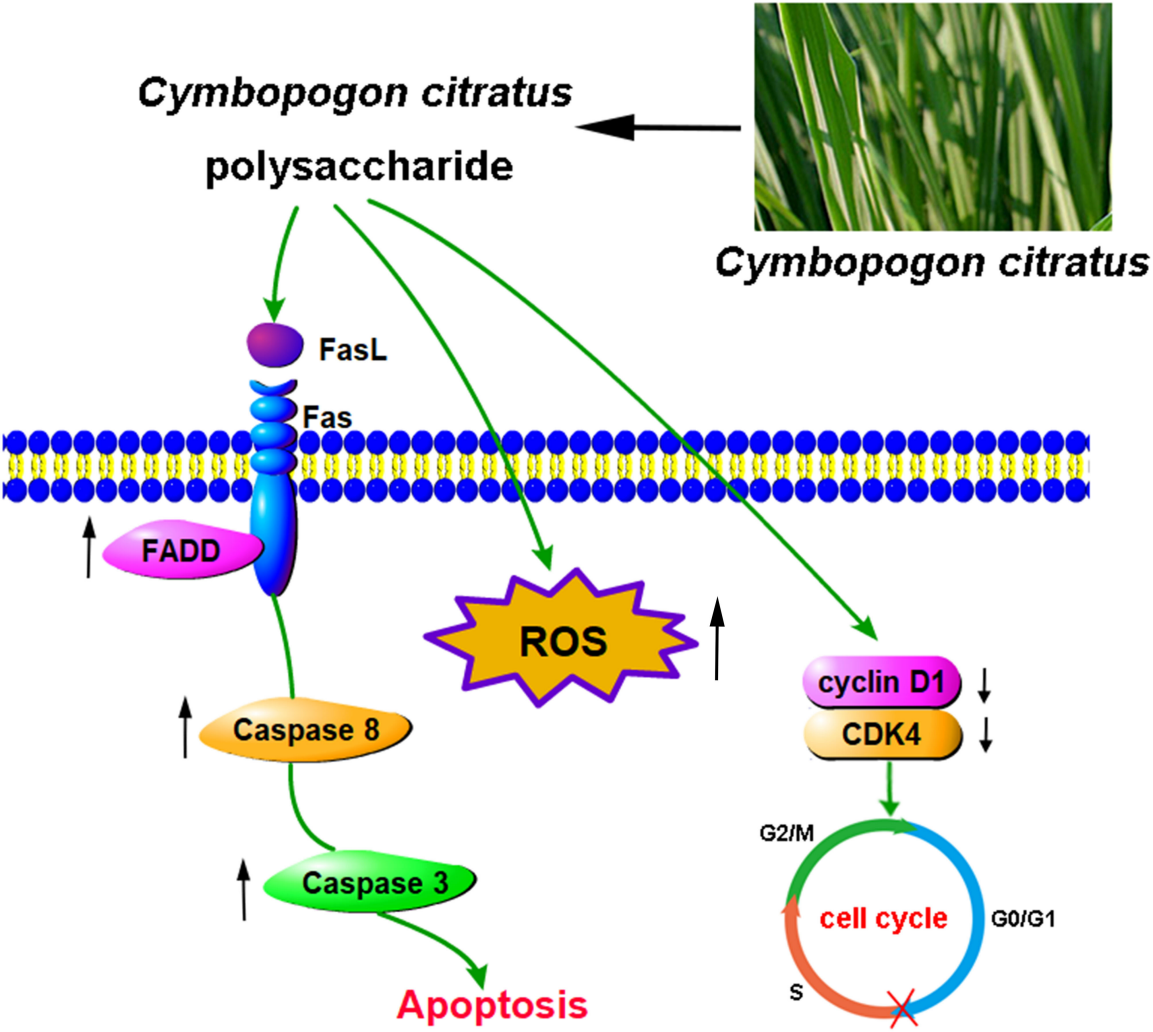
The proposed molecular mechanisms of cell cycle arresting and apoptosis induced by CCP in MDA-MB-231 cells.

The death receptor pathways, including TNFR, TRAIL and Fas/FasL signaling pathways, are one of the most important pathways of cell apoptosis (51). The Fas/FasL system played an important role in maintaining cell colonies, clearing malignant transformed cells and regulating the immune system (52). Fas, a type I cell surface glycoprotein, belongs to the cytokine receptor family, which is similar to tumor necrosis factor (TNF) receptors (53). FasL is a type II across a membrane protein on the surface of the cell, belonging to the tumor necrosis factor (TNF) family (54). When the ligand FasL binds to the Fas receptor, it attracts FADD with the same death domain in the cytoplasm. Subsequently, FADD can recruit procaspase-8 through its death effector domain (DED). Once caspase-8 is activated, it cleaves and activates the key downstream apoptotic executive factor caspases-3 to induce apoptosis (55). According to the results of this study, CCP treatment significantly increased the level of activated Caspase-8/-3. Furthermore, the expression of Fas, FasL, FADD in MDA-MB-231 cells were significantly increased, suggesting that CCP-induced apoptosis could be triggered through Fas/FasL-mediated death receptor pathway in MDA-MB-231 cells (Figure 8). In consistent with our results, the antitumor activities of *Gracilariopsis lemaneiformis* Polysaccharide was associated with apoptosis-related Fas/FasL signaling pathway in the human lung cancer cell line A549, the gastric cancer cell line MKN28, and the mouse melanoma cell line B16 (56).

## Conclusions

Collectively, CCP was a high molecular weight polysaccharide extracted from *C. citratus*. According to the results of HPGPC, IC, FT-IR, methylation and NMR, CCP was a 1.98 × 10^6^ Da acidic polysaccharide with a complex structure mainly composed of Gal, Ara, Glc and Rha. In MDA-MB-231 cells, the proliferation was significantly inhibited after CCP treatment. Moreover, we found that the G_0_/G_1_ arrest was induced by CCP in a dose-dependent manner, and Fas/FasL-mediated death receptor pathway was thought to participate in the process of CCP-induced apoptosis. These experimental results demonstrated that *C. citratus* polysaccharide could potentially be used as an easily available natural source for breast cancer therapy, which can be widely used in the food industry in the future.

## Data Availability Statement

The raw data supporting the conclusions of this article will be made available by the authors, without undue reservation.

## Author Contributions

YC and HL designed the whole study. YC and SQ carried out all experiments. SQ, YC, and HX acquired and interpreted data. YC wrote the original version of the manuscript. HL and PC revised and edited the manuscript. All authors contributed to the article and approved the submitted version.

## Funding

This research work was funded by the Key Research and Development Project of Shanxi Province (Grant No. 201903D211008), the Natural Science Foundation of Jiangsu Province (Grant No. BE2019351), and the Natural Science Foundation of Tianjin City of China (Grant No. 21YDTPJC00060).

## Conflict of Interest

The authors declare that the research was conducted in the absence of any commercial or financial relationships that could be construed as a potential conflict of interest.

## Publisher's Note

All claims expressed in this article are solely those of the authors and do not necessarily represent those of their affiliated organizations, or those of the publisher, the editors and the reviewers. Any product that may be evaluated in this article, or claim that may be made by its manufacturer, is not guaranteed or endorsed by the publisher.
